# The future of telemedicine visits after COVID-19: perceptions of primary care pediatricians

**DOI:** 10.1186/s13584-020-00414-0

**Published:** 2020-10-20

**Authors:** Zachi Grossman, Gabriel Chodick, Stephen M. Reingold, Gil Chapnick, Shai Ashkenazi

**Affiliations:** 1 Adelson School of Medicine, Ariel University, Ariel, Israel; 2 Pediatric Clinic, Maccabi Healthcare Services, 26 Rofe Hamachtarot, 69372 Tel Aviv, Israel; 3 Maccabitech, Maccabi Healthcare Services, Tel-Aviv, Israel; 4 Sackler Faculty of Medicine, Tel-Aviv University, Tel-Aviv, Israel; 5 Meuhedet Health Maintenance Organization, Modi’in, Israel; 6 Maccabi Healthcare Services, Kfar Saba, Israel

**Keywords:** COVID-19, Telemedicine, Pediatricians

## Abstract

**Background:**

Facing the global health crisis of COVID-19, health systems are increasingly supporting the use of telemedicine in ambulatory care settings. It is not clear whether the increased use of telemedicine will persist after the pandemic has resolved. The aims of this study were to assess the use of telemedicine by Israeli pediatricians before and during the first lockdown phase of the pandemic, and to elucidate how they foresee telemedicine as a medium of medical practice in the post-pandemic era.

**Methods:**

A web-based survey was distributed among Israeli pediatricians in May 2020, soon after the end of first lockdown was announced. The survey assessed the frequency of telemedicine use as well as its influence on clinical decision making before and during the first COVID-19 lockdown, using two hypothetical clinical scenarios. The same scenarios were also used to assess how the pediatricians foresaw telemedicine in the post-pandemic period. In addition, administrative data from Maccabi on telemedicine use before, during and after the first lockdown were retrieved and analyzed.

**Results:**

One hundred and sixty-nine pediatricians responded to the survey (response rate = 40%). The percentage of respondents who reported daily use of text messages, pictures and videoconferencing increased from 24, 15 and 1% before COVID-19 to 40, 40 and 12% during the lockdown, respectively (*p* < 0.05). After the pandemic, projected use of text messages and pictures/videoclips was expected to decrease to 27 and 26% of respondents, respectively (*p* < 0.05), but pictures/videoclips were expected to increase from 15% of respondents before to 26% of respondents after (*p* < 0.05). The reported high likelihood of treating suspected pneumonia or prescribing antibiotics for suspected otitis media via telemedicine was expected to decrease from 20% of respondents during the COVID-19 lockdown to 6%% of respondents after (*p* < 0.05), and from 14% of respondents during the lockdown to 3% of respondents after, respectively. (*p* < 0.05).

Maccabi administrative data indicated that during the lockdown, there was an increase in phone visits and a decrease in in-person visits compared to the pre-lockdown levels of use. One month after the end of the first lock-down there was a partial return to baseline levels of in-person visits and a sustained increase in phone visits. Phone visits accounted for 0% of pediatrician visits before the first lockdown, 17% of them during the lockdown, and 19% of them 1 month after the lockdown relaxation.

**Conclusions:**

The study indicates that use of telemedicine technologies by primary care pediatricians increased substantially during the first COVID-19 lockdown. The study also found that pediatricians expected that use levels will recede after the pandemic. As the pandemic continues and evolves, it will be important to continue to monitor the level of telemedicine use as well as expectations regarding post-pandemic use levels.

## Background

Telemedicine (or telehealth), is defined by the National Institutes of Health (NIH) as the use of technology to provide and support healthcare at a distance [1]. One of the uses of telemedicine is the establishment of a remote link between doctors and their patients. Telemedicine does not necessarily replace the in-person visit; it could be an addition to the in-person visit. The advantages of such a remote link include enhanced access (virtual visits in addition to frontal visits) in which personal physicians may provide improved medical care for children, and as such, is supported by the American Academy of Pediatrics [2]. This context permits providers access to the complete medical record and an established relationship with the patient, both of which are conducive to making appropriate care decisions [2]. Telehealth technologies run the gamut from telephones and text messages, to transmitted images, and on to real-time videoconferencing [3]. Providers who frequently use these tools to deliver health care tend to be early adopters of new technology [4].

Throughout most of 2020, the world has been facing a global health crisis: the COVID-19 pandemic [5]. Although the focus of tackling the direct impact of COVID-19 occupies the primary focus of many health organizations, the maintenance of core and critical clinical service is of no less importance. In many countries, the initial reaction was for healthcare facilities to reduce or even cease many clinical services, including the closure of clinics and the postponement of all non-critical medical services [6]. However, telemedicine affords continued medical care while adhering to strict social distancing. Patients at risk may benefit from staying at home, thereby reducing exposure to others, while continuing to receive medical care [7]. Therefore, it is no surprise that health systems worldwide are resorting to telemedicine, resulting in an exponential increase in telemedicine use, contrasting to a previously slow adoption of the new practice [8]. In the US [9], UK [10] and Israel [11], COVID-19 guidelines support the use of telemedicine in ambulatory care settings for children during the pandemic.

The two most crucial considerations in the current situation are the quality of the telemedicine service delivered and its safety [12]. Some of the efforts to promote telemedicine during the pandemic appears to assume a that a sizeable proportion of outpatient visits can be managed effectively remotely, and patients can be triaged to telemedicine services without compromising their safety or the quality of care. It is not clear, however, whether this is true. It is also not clear whether the utility of tele-triage will dissipate, in part or in total, after the pandemic crisis is over [8].

The aim of the study is to collect preliminary data on telemedicine experiences by Israeli pediatricians during the early phase of the COVID-19 pandemic as well as their expectations at that time regarding future use after the pandemic resolves. The objective of the data collection is to ascertain community physicians’ attitudes, experiences and medical decision making practices while using telemedicine during a period in which physical contact was limited, and how the extent to which they foresaw telemedicine as a medium of ideal medical practice when the crisis will be over. The latter will also help characterize the provider who tends more to telemedicine practices as compared to peers.

The data collection will evaluate the pediatricians’ confidence in managing diseases remotely in two hypothetical clinical scenarios. We will discuss these findings and discuss how the boost in telemedicine during the COVID-19 pandemic is likely to affect the practice of telemedicine the day after the crisis and shape the future of health care delivery.

## Methods

### Study sample

Structured questionnaires were distributed to Israel Pediatric Research in Office setting NETwork (IPRONET) members in May 2020, soon after the relaxation of Israel’s first lockdown was announced. The Israel Pediatric Research in Office Setting (IPROS) network was established in 1995 and includes Israeli pediatricians willing to collaborate with the network on performing research in their clinics [13]. The IPROS Network mailing list, IPRONET, is an electronic mailing list that can be joined voluntarily by all pediatricians in Israel. Initially it was established as an interest group intended to promote research, but as the years went by it became more like an active forum of pediatricians. As such, it is now more like an open forum, a place where research proposals are still being discussed but where the focus has shifted to other issues relating to child health in Israel - policy controversies, clinical dilemmas and also information on upcoming conferences. A call to join IPRONET is sent to all registered Israeli pediatricians (currently 2700) annually. A few family physicians have also joined the network throughout the years.

### Representativeness and comparability

The general characteristics of the study participants (age, gender) were compared to those of all Israeli pediatricians as published in Israel’s Ministry of Health (IMOH) report on health professionals in 2018 [14], the most current database available.

### Research instrument

#### Socio demographic details

Participants were asked about gender, age, years in practice, place of work (community clinic, hospital), Health Fund employment, and specialty.

#### Use of telehealth technologies

Respondents were asked about the frequency of use of the following telehealth technologies: phone calls, emails, text messages via mobile phone, pictures or video clips via mobile phone, video-conferencing via Zoom or Facetime, and Tytocare or American Well digital remote devices. Tytocare offers a medical device that can function as an otoscope and stethoscope, allowing for the remote examination of the ears, throat, skin, heart, and lungs. American Well is a video session that is integrated into the patients’ EMR (electronic medical record) and permits the uploading of documents and photos. (The latter two technologies are available in only some of the Health Funds). The study assessed the pattern of using these technologies before and during the COVID-19 pandemic, as well as projected future use of these technologies after the pandemic resolves. The responses were evaluated using a 5-point Likert scale ranging from “Almost never used” to “Used at all times”. The availability of access to the child’s medical record while providing tele-practice was evaluated, ranging from “full access” to “no access.”

#### Clinical scenarios

Two hypothetical scenarios were posed to evaluate likely clinical decision making by the respondents. The first scenario, of a suspected pneumonia, was presented as, *“The parents of a seven-year-old girl contact you and report that the child has had 4 days of high fever, cough, and nasal congestion. The child is in not in distress, has mild anorexia, no vomiting and passed 2 loose stools today.”* The respondents were asked to rate the likelihood of possible use of the following treatments or diagnostic testing choices: prescribing antibiotic treatment, prescribing symptomatic treatment, referring to an emergency room, requesting for a chest X-ray, and prescribing corticosteroids in case of wheezing.

The second scenario, of a suspected otitis media, was presented as, “*The parents of a two-year-old boy contact you and report that the child has had fever for 2 days, mild upper respiratory symptoms and left ear pain. The child is vigorous, eating well, had one loose stool, but slept poorly last night*.” Like in the first scenario, the likelihood of the possible use of various treatments was evaluated: prescribing antibiotic treatment and prescribing symptomatic treatment.

In both scenarios the respondents were also asked to rate the likelihood of treating such cases via telemedicine based on the assumptions that the patient is known to the respondent, and that there is no suspicion of a COVID-19 case. It is worth mentioning that when the survey was conducted, the definition of a suspected COVID − 19 case was based on strict supportive epidemiologic criteria [15], unlike the definition used subsequently during the second wave. The questions regarding the scenarios related to decision making while the respondents provide telemedicine in three different periods of time: before COVID-19, during the pandemic and a future perspective on decision making when the pandemic is over. The responses were evaluated using a 5-point Likert scale ranging from “absolutely unlikely” to “always likely”.

#### Maccabi administrative database

To learn more about the pattern of telehealth use, we analyzed Maccabi Healthcare Services administrative data for use of pediatricians’ in-person and phone visits. Maccabi Healthcare Services has 2.4 million patients, representing 25% of the Israeli population. The data on pediatricians’ visits is generated by 550 pediatricians who provide care to Maccabi’s children. In person visit is defined as a visit where the parents and the child physically attended the pediatrician’s clinic. Phone visit is defined as a scheduled phone call with the pediatrician without attending the clinic. We analyzed these visits in three periods of time, each 45 days long: prior to the first COVID lockdown (February 1 to March 15), during the lockdown (March 15–April 30), and after lockdown relaxation (May 1 – June 15).

### Data analysis

Chi-square tests evaluated differences in utilization of telemedicine modalities between categorical groups. Physicians reporting on plans to utilize pictures/videoclips or videoconferencing several times daily after the pandemic were defined. Binary logistic regression analysis (enter method) was utilized to assess the relation between their status (dichotomized dependent variable) and the independent variables, including personal characteristics such as professional group, gender (female vs. male), as well as scores on adoption of telemedicine prior to the COVID-19 outbreak in the study questionnaire. Age and years of experience were examined as independent variables. The odds ratio is per 1 year of age. We reported odds ratios (OR), 95% confidence intervals (CI) results of the best fitting adjusted regression models. Two-sided level of significance was set to *p* < 0.05. A comparison of the mean daily number of phone and in-person physician visits in Maccabi in the periods before COVID 19 lockdown, during lockdown and thereafter was performed. All statistical analyses were conducted in IBM-SPSS Statistics for Windows (Version 25.0. Armonk, NY, IBM Corp.).

## Results

### Survey findings

Of 423 IPRONET members, 169 pediatricians responded to the survey, for a response rate of 40%. Demographic characteristics of the study sample are presented in Table 1. The characteristics of the study participants were similar to those of all Israeli pediatricians [14], regarding age groups distribution (45% under 55 years in study participants vs. 49% in the IMOH report, *p* = 0.332) and gender (51% females in the study vs. 53% in the report, *p* = 0.216).

**Table 1:** Characteristics of the study sample

	n	%
Age (years)		
25–44	47	27.8
45–54	45	26.7
55–66	52	31.0
67–74	25	14.4
Gender		
Male	82	48.5
Female	87	51.5
Specialty		
Pediatrics	165	97.6
Family medicine	3	1.8
General	1	0.6
Clinical experience post-residency (years)		
< 10	29	17.2
11–20	51	30.2
21–30	51	30.2
> 30	38	22.5
Works also in a hospital		
Yes	42	24.9
No	127	75.1
Health fund employment		
Clalit	58	34.3
Maccabi	43	25.4
Meuhedet	24	14.2
Leumit	9	5.3
More than one Health fund	35	20.8

### Use of telehealth technologies

Fifty six percent of the respondents reported full access to the child’s medical record in more than 75% of telehealth visits, while only 9% reported no access to the record during such visits.

Table 2 presents the respondents’ daily use of telemedicine technologies (phone calls, emails, text messages, pictures/video-clips, and videoconferencing) before and during the first COVID-19 lockdown period (hereafter “the COVID-19 lockdown”) and projected use after the pandemic will have ended.

**Table 2:** Daily use pattern of technologies

Technology	Respondents					
	Before COVID-19		During COVID-19		After COVID-19	
	n	%	n	%	n	%
Phone calls	81	47.9	110	65.1^*^	99	58.6
Emails	41	24.3	73	43.2^*^	57	33.7
Text messages	40	23.7	67	39.6^*+^	45	26.6
Pictures/video-clips	25	14.8	68	40.2^*+^	44	26.0^#^
Video – conferencing (Zoom, Facetime)	2	1.2	21	12.4^*^	9	5.3

^*^During vs. before, *p* < 0.05

^+^During vs. after, *p* < 0.05

^#^After vs. before, *p* < 0.05

Reported daily use of all technologies significantly increased during the COVID-19 lockdown. The projected use of text messages and pictures/videoclips after the pandemic will have ended is projected to significantly decrease in relation to the pandemic period. However, the use of pictures/videoclips is projected to significantly increase after the pandemic compared to pre-COVID- 19 use.

Tytocare digital remote device use has been reported by 3 and 6% of respondents for before and during the COVID- 19 lockdown, respectively. Similarly, American Well use was reported by 5 and 8% of respondents, before and during the COVID- 19 lockdown, respectively. (Data not shown in Table 2).

### Scenario 1

Patterns of clinical decision making via telemedicine before, during and after COVID-19 lockdown for Scenario 1 are presented in Table 3. The reported high likelihood pattern of the following- treating the patient via telemedicine, prescribing symptomatic treatment, referring to an emergency room, prescribing steroids and requesting for a chest X-ray – significantly increased during the pandemic lockdown. However, after the pandemic, the respondents expect the likelihood of these treatments to decrease significantly. Similarly, the reported medium likelihood pattern of prescribing antibiotics significantly increased during the pandemic lockdown but is projected to decrease after the pandemic dissipates.

**Table 3:** Likelihood patterns of using different treatments or diagnostic testing choices in scenario 1

Likelihood pattern	Respondents					
	Before COVID-19		During the first COVID-19 lockdown		After the first COVID-19 lockdown	
	n	%	n	%	n	%
Treating via telemedicine High likelihood	7	4.1	33	19.5^*+^	10	5.9
Prescribing antibiotics Medium likelihood	4	2.4	30	17.8^*+^	9	5.3
Prescribing symptomatic treatment High likelihood	18	10.7	45	26.6^*+^	20	11.8
Referring to emergency room High likelihood	15	8.9	38	22.5^*+^	12	7.1
Prescribing steroids if wheezes High likelihood	14	8.3	34	20.1^*+^	16	9.5
requesting for chest X ray High likelihood	20	11.8	40	23.7^*+^	21	12.4

^*^During vs. before, *p* < 0.05

^+^During vs. after, *p* < 0.05

### Scenario 2

Patterns of clinical decision making via telemedicine for Scenario 2 before, during and after the COVID-19 lockdown are presented in Table 4. The reported high likelihood pattern of the following- treating the patient via telemedicine, prescribing antibiotic treatment, prescribing symptomatic treatment– significantly increased during the pandemic lockdown. However, after the pandemic will have ended, the reported future high likelihood pattern of these decisions is significantly projected to decrease comparing to the pandemic period.

**Table 4:** Likelihood patterns of using different treatments in scenario 2

Likelihood pattern	Respondents					
	Before COVID-19		During the first COVID-19 lockdown		After the first COVID-19 lockdown	
	n	%	n	%	n	%
Treating via telemedicine High likelihood	18	10.7	70	41.4^*+^	23	13.6
Prescribing antibiotics High likelihood	3	1.8	23	13.6^*+^	5	3.0
Prescribing symptomatic treatment High likelihood	43	25.4	67	39.6^*+^	39	23.1

^*^During vs. before, *p* < 0.05

^+^During vs. after, *p* < 0.05

In a univariate model, pre-pandemic use of pictures/videoclips or videoconferencing during visits was the only significant (*p* < 0.001) predictor of frequent provision of these technologies after the pandemic. Physicians who reported occasional and frequent pre-pandemic use of pictures/videoclips or videoconferencing during visits had odds ratios of 2.73 (95% CI:1.01–7.42) and 82.24 (95% CI: 17.50–386.58) respectively for frequent post-pandemic provision of these technologies, as compared to physicians who did not use it.

#### Findings from the administrative data

**Fig. 1:**
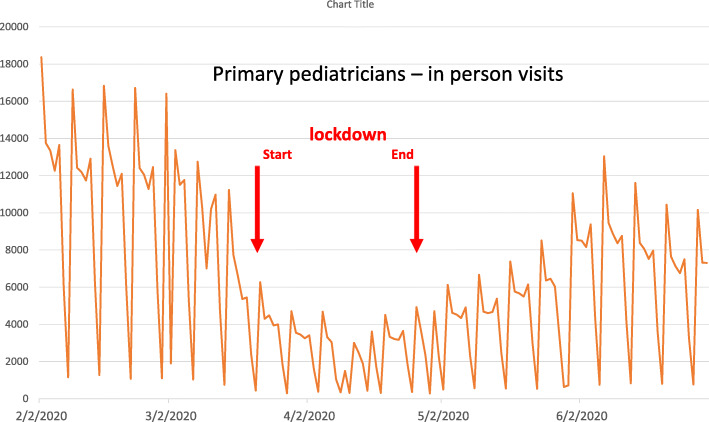
Pediatric frontal visits

**Fig. 2:**
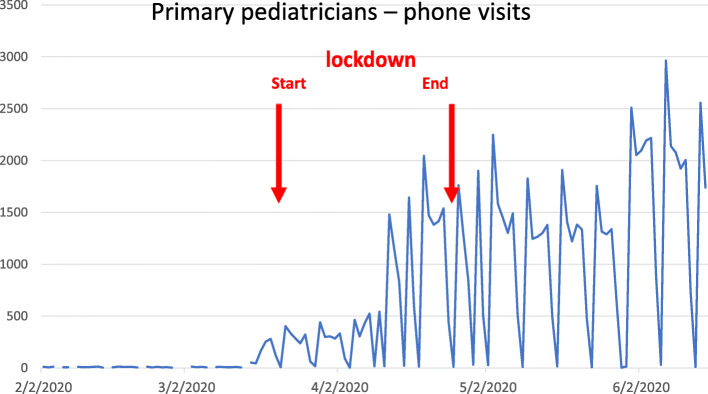
Pediatric phone visits

Figures 1 and 2 present phone and in-person visits data for Maccabi Healthcare Services, based on data from Maccabi’s patient database, regarding the period before the COVID 19 lockdown, during the lockdown and afterwards. A drop in in-person visits during the first lockdown (mid-March to end of April), and a partial return in relaxation period to pre-lockdown level are shown in Fig. 1. A sharp increase in phone visits during the lockdown, sustained after the lockdown was eased is presented in Fig. 2. Comparison of number of visits and changes at the different periods is presented in Table 5.

**Table 5:** Comparison of visits data at selected periods

Visit type	Visits data				
	Mean daily number pre lockdown (1/2/2020–15/3/2020)	Mean daily number lockdown (15/3/2020–30/4/2020)	% Change (lockdown compared to pre lockdown)	Daily number post lockdown relaxation (1/5/2020–15/6/2020)	% Change (post lockdown relaxation compared to lockdown)
In- person	9723	2978	−70*	5420	+ 82*
phone	9	575	+ 5952*	1279	+ 122*

*p = 0.002

Data on the number and mix of visits (in person and phone) in the three periods – pre lockdown, during lockdown and post lockdown) are presented in Table 6. Total pediatrician visits decreased from 9.7 thousand in the first period to 3.5 thousand in the second period, and then increased to 6.7 thousand in the third period. Between the second and third periods both in-person visits increased by 82%, and phone visits increased by 122%. Phone visits accounted for 0% of pediatrician visits before the pandemic, 17% of them during the first lockdown period, and 19% of them 1 month after the lockdown.

**Table 6:** Visit mix in the three periods

Visit type	Mean daily number of visits and percentage of total visits					
	Pre first lockdown period		First lockdown period		Post lockdown relaxation period	
	number	Percentage %	number	Percentage %	number	Percentage %
Total visits	9733	100	3553	100	6699	100
In-person visits	9723	100	2978	83	5420	81
Telemedicine visits	9	0	575	17	1279	19

We did not find any statistically significant inter-regional differences.

## Discussion

Our results confirm the rise in use of all evaluated telemedicine technologies during the first COVID-19 lockdown in Israel. Physicians complied with pandemic-era guidelines of health organizations world-wide in delivering pediatric care via telemedicine when feasible [9–11].

However, even though physicians predict that they will revert to using fewer text messages, digital photos and video clips after the pandemic has ended, the rate of projected use of photos and clips is higher than pre-COVID-19 levels, suggesting that a projected increase will be sustained for the use of the latter modalities.

When evaluating the clinical decisions made in the two hypothetical scenarios, the high likelihood pattern of treating the patient via telemedicine was significantly increased during the pandemic and projected to decrease after the pandemic. This pattern was also observed while exploring individual clinical decisions – prescribing antibiotic treatment and prescribing symptomatic treatment.

These observations are in accordance with existing evidence regarding physicians’ attitudes and perceived barriers to telemedicine. One of the biggest barriers to the use of telehealth has been the resistance on the part of providers to embrace this technology in caring for patients [16]. In a study exploring the experiences, attitudes and challenges of physicians in a pediatric telemedicine service operated in Israel, it was demonstrated that physicians face a variety of challenges, requiring special expertise, qualities, and skills [17]. The main factor the doctors mentioned was the difficulty in making a diagnosis from a distance, which is attributed to the fundamental difference between the virtual visit and the in-person visit, due to the doctor’s inability to perform a physical exam in the telemedicine setting. Unlike the traditional encounter between patient and doctor, in the telemedicine format the doctor learns about the patient’s medical problem from his verbal description, usually without being able to physically examine him [17]. A study of telephone triage reported patient safety risks and the lack of visual clues which aid physicians in identifying patients in acute condition [18].

Considering these observations, it is not surprising that most of our study respondents viewed telemedicine during the first COVID-19 lockdown as a temporary solution for a distinct period that required social distancing. When routine care resumes, our respondents, for the most part, preferred to return to traditional in-person care. The dynamics of the use of in-person visits as demonstrated by the administrative data (a sharp decrease during lockdown and a partial return to baseline shortly after) supports our survey results. The sustained increase in phone visits after lockdown relaxation probably reflects parental preferences to phone visits facing the continuing policy of social distancing even after relaxation.

In addition, the administrative data documents that the total pediatric visits decreased from March onwards. This was probably due to two reasons: first, spring and summer (spring more than summer) are usually characterized by less pediatric morbidity and second, masks and partial school and kindergarten closures could have reduced the burden of communicable diseases other the COVID 19. The mix of visits from the lockdown onwards is the same as in the lockdown itself, probably because we are still basically practicing COVID time daily routine, and COVID social distancing policy still prevails. Parents try to avoid even now coming to the clinics if not necessary, which keeps on the relatively high demand for phone visits.

As of yet, there is no valid evidence regarding either parental perspectives on pediatric telemedicine care delivered during the COVID-19 pandemic, or their attitudes on the future of this modality once the pandemic will be over. Once the lockdown restrictions were lifted in Israel, face-to-face pediatric visits increased rapidly (personal communication), suggesting that parents prefer in-person visits. Again, this feeling is supported by our administrative data demonstrating partial return to baseline level of in-person visits. Cautious speculation may suggest that parents view telemedicine as a viable, but temporary solution.

Using telehealth to submit pictures and video-clips may continue after the pandemic, as predicted by respondents. The use of pictures is prevailing mostly in pediatric dermatology. The universal availability of cell phone cameras and the ease of digital communication have dramatically increased this type of consultation – tele-dermatology. Studies have demonstrated that most skin lesions in pediatric primary care attention could be managed by tele-dermatology [19, 20]. It appears that pictures and videoclips have clear advantages over other technologies both for physicians and for parents; unlike other technologies, they are transmitting relevant and accurate clinical information in a matter of seconds.

The reported use of remote diagnostic devices such as Tytocare was negligible. This device has been recently proven to provide high quality sound and images, on par with traditional medical devices [21]. Yet, to the best of our knowledge, the validity of its use by parents has not been demonstrated in a peer reviewed journal publication. Currently, Tytocare is included in the service delivery in only one Health Fund (Clalit), and has a limited number of users, presumably restricted due to its cost. This may have contributed to its low use in our study.

In both clinical scenarios, there was a rise in the likelihood of antibiotic prescribing during the pandemic. The risk of over-prescription of antibiotics in telemedicine visits has been previously studied concerning both adults and children. A study of adult patients using direct to consumer (DTC) telemedicine compared with physician office visits has identified more inappropriate antibiotic prescriptions for bronchitis as well as broader spectrum antibiotic use [22, 23]. In another study, children with acute respiratory tract infections (ARTI) managed with DTC were less likely to receive guideline-concordant antibiotic management compared to children at primary care pediatrician visits [24]. Inappropriate antibiotic prescribing, partly to satisfy consumers, can harm patients through the emergence of antibiotic resistance, adverse drug effects and disruption of the microbiome.

In light of these considerations, the American Academy of Pediatrics discourages the use of DTC telemedicine outside the medical home and suggests that DTC telemedicine should not be used for children < 2 years old. The medical home, where the physician is familiar with the patient and has access to the medical chart, is the preferred setting for delivery of care, thus minimizing the risk of inappropriate antibiotic prescribing [25]. In our study, 56 % of the respondents reported full access to the child’s medical record in more than 75% of the telehealth visits, and in the hypothetical scenarios respondents were asked to assume that the patients were known to the provider. Yet, as our study has demonstrated, the projected likelihood of antibiotic prescribing via telemedicine after the pandemic resolves, is significantly decreased, in correlation with physicians’ preference to in-patient visits. It is possible that in general, pediatricians prefer frontal ARTI visits to avoid inappropriate antibiotic treatment. This may reflect a component of the “moral conflict” that has been reported by pediatricians in an Israeli study on a pediatric telemedicine service [17] between maintaining standard of care and pleasing parents.

The characteristics of providers who adapt to the use of telemedicine tools more so than their peers have been previously described [4, 26, 27]. Based on our study’s findings, we define such a provider as the physician who predicts using teleconferencing and/or digital images several times per day with their patients, even after the pandemic resolves. According to our results, such a provider is the physician who adopted pre pandemic frequent use of the new technologies. In his 1962 book, *Diffusion of Innovations*, Rogers defines such individuals as “early adopters,” customers who are the first to utilize new technologies [28].

It is assumed that the practice of most physicians will evolve to integrate an increasing number of elements of virtual care. As such, medical education and training will need to adapt, preparing tomorrow’s physicians for the environment in which they will be practicing [25]. It will be necessary for early adopters, together with medical specialty societies, to formalize the curriculum and training [4].

Reimbursement for telemedicine is not standardized. In the US, it varies by location, services provided, and payers. Individual states’ laws vary concerning telehealth licensing, reimbursement, and consent, as well as the very definition of telehealth. Currently, there is no set standard for private health insurance providers regarding telemedicine. As for Medicare beneficiaries, telemedicine visits are considered the same as in-person visits and compensate both at the same rate [29]. To our knowledge, in Israel, medical services provided via telemedicine are compensated the same as regular in-person visits.

Our study has several limitations. First, the study was potentially biased upstream of the incomplete response rate, with 84% of pediatricians declining to join IPRONET despite annual solicitation.

Second, the study focused on a sample of Israeli pediatricians, and due to the incomplete response rate (40%) the sample may not be representative of all Israeli pediatricians, resulting in bias. We therefore recommend replicating the study in a variety of settings, such as to all pediatricians in one or more of the Health Funds. To compensate for these limitations, we compared characteristics of the study participants with available characteristics of Israeli pediatricians as reported by the Israel Ministry of Health and confirmed no significant differences.

Third, the survey data regarding use of telemedicine technologies before and during COVID-19 was subjective; perceived level of use may not reflect the actual use. We partially compensated for this limitation by adding frontal and phone visit administrative data. Still, studies are warranted in which technology use is assessed by more objective means such as electronic health record data and smartphone use data.

Fourth, while exploring phone calls we did not distinguish in the survey between regular phone calls (as done in the pre COVID − 19 era) and reimbursed “phone visits”. Therefore, we cannot rule out the possibility that physicians would like to continue with this kind of a visits in the post COVID-19 era.

Fifth, the results of the scenarios are dependent on the characteristics of the scenarios. Since both scenarios were cases where physical examination is crucial, the physicians answered as described. They might have answered in another way had the scenarios been different. Sixth, in the scenarios, we focused only on likelihood of using telemedicine technologies as grounds for clinical management. In future studies, however, physicians’ assessment of the appropriateness of these technologies and their comfort using them should be explored.

Seventh, the data in our study is relevant to May 2020, soon after the first lockdown relaxation was announced. As the pandemic continues and evolves, it will be important to continue to monitor the level of telemedicine use as well as expectations regarding post-pandemic use levels. On the one hand, the longer the duration of the COVID-driven push to substitute virtual visits for in person visits, the more likely it is that virtual visits will become the new normal that will continue even after the pandemic has ended. On the other hand, if parents will prefer in-person visits once the pandemic is over, as these visits may better provide them with the holistic nature of the doctor-patient relationship, then virtual visits will be mainly used for dermatologic conditions where pictures can easily convey the clinical information, or for conditions where parents cannot technically attend the clinic. Only time, and continued monitoring, can determine which of these scenarios will play out in practice.

## Conclusions

Our study highlights the rise in use of telemedicine technologies during the first COVID-19 lockdown in Israel, as well as its projected partial decline in use after the pandemic has ended. Pictures and videoclips may have a potential role as part of the service delivery in the future. It is clearly demonstrated that, as of May 2020, most Israeli pediatricians preferred, once the pandemic subsides, to revert to in-person visits and base their clinical decisions on frontal data rather than on data gathered by telemedicine encounters. This preference is deeply inherent in physicians’ education, regarding the roles of the physical encounter and the importance of the physical examination.

Yet, as the practice of most physicians will evolve to integrate an increasing number of elements of virtual care, it is of utmost importance to prepare the workforce for this evolution. We have identified in our study a group of physicians who prefer the frequent use of telemedicine services, even after the pandemic resolves. These physicians have been characterized in general as early adopters of technology. They should possibly be considered for teaching roles in pediatric education, and together with pediatric societies, prepare future pediatricians for the virtual evolution.

As the pandemic continues and evolves, it will be important to continue to monitor the level of telemedicine use as well as expectations regarding post-pandemic use levels.

## Data Availability

The datasets used and/or analysed during the current study are available from the corresponding author on reasonable request.

## Abbreviations

IPRONET: Israel Pediatric Research in Office Setting Network
COVID-19: Coronavirus Disease 2019
NIH: National Institute of Health
IPROS: Israel Pediatric Research in Office Setting
IMOH: Israel Ministry of Health
EMR: Electronic medical record
OR: Odds Ratio
CI: Confidence Interval
DTC: Direct to Consumer
ARTI: Acute Respiratory Tract Infections
